# Protein C: a potential biomarker in severe sepsis and a possible tool for monitoring treatment with drotrecogin alfa (activated)

**DOI:** 10.1186/cc6854

**Published:** 2008-04-04

**Authors:** Andrew F Shorr, David R Nelson, Duncan LA Wyncoll, Konrad Reinhart, Frank Brunkhorst, George Matthew Vail, Jonathan Janes

**Affiliations:** 1 Department of Medicine, Section of Pulmonary and Critical Care Medicine, Washington Hospital Center, Irving Street, Washington, District of Columbia 20010, USA; 2 Lilly Research Laboratories, Eli Lilly and Company, 520 S. Meridian, Indianapolis, Indiana 46285, USA; 3 Department of Critical Care, Guy's and St Thomas' NHS Foundation Trust, Lambeth Palace Road, London SE1 7EH, UK; 4 Department of Anesthesiology and Intensive Care, Friedrich Schiller University, Erlanger Allee, Jena 07740, Germany

## Abstract

**Introduction:**

Drotrecogin alfa (activated; DrotAA) treatment, a 96-hour infusion, reduces 28-day mortality in severe sepsis to approximately 25%. The question remains whether a longer infusion or higher dose could increase rate of survival. The goal of this study was to identify a dependable, sensitive measure with which to monitor disease progression and response in patients during DrotAA treatment.

**Methods:**

Data on severe sepsis patients included in PROWESS (placebo-controlled, double-blind, randomized study of 850 DrotAA and 840 placebo individuals) and ENHANCE (single-arm, open-label study of 2,375 DrotAA patients) studies were analyzed. In these studies, DrotAA (24 μg/kg per hour) or placebo was infused for 96 hours and patients were followed for 28 days. Data on six laboratory measures and five organ dysfunctions were systematically analyzed to identify a potential surrogate end-point for monitoring DrotAA therapy and predicting 28-day mortality at the end of therapy. To allow comparison across variables, sensitivity and specificity analyses identified cut-off values for preferred outcome, and relative risks for being above or below cut-offs were calculated, as was the 'proportion of treatment effect explained' (PTEE) to identify biomarkers that contribute to benefit from DrotAA.

**Results:**

Protein C was the only variable that correlated with outcome across all analyses. Using placebo data, a baseline protein C under 40% was established as a useful predictor of outcome (odds ratio 2.12). Similar odds ratios were associated with cut-off values of other biomarkers, but the treatment benefit associated with DrotAA was significantly greater below the cut-off than above the cut-off only for protein C (relative risk for 28-day mortality 0.66 versus 0.88; *P *= 0.04). Protein C was the only end-of-infusion biomarker that potentially explained at least 50% of the benefit from DrotAA (PTEE 57.2%). The PTEE was 41% for cardiovascular Sequential Organ Failure Assessment score and for d-dimer. At the end of infusion (day 4), protein C categories (≤40%, 41% to 80%, and > 80%) remained significantly related to mortality, regardless of treatment assignment.

**Conclusion:**

Based on systematic analyses of 11 variables measured in severe sepsis clinical trials, protein C was the only variable consistently correlated with both DrotAA treatment effect and survival. Further study is needed to determine whether longer infusions or higher doses of DrotAA would achieve the goal of normalizing protein C in more patients with severe sepsis.

## Introduction

Biomarkers play an important role in clinical care [1,2]. Biomarkers facilitate diagnosis, aid in assessing the severity of disease, and provide clinicians with surrogates that they can follow to assess response to therapy. In a number of areas, biomarkers are critical in the management of complex disease states. For example, brain natriuretic peptide is now routinely measured in patients suspected of having decompensated congestive heart failure [3,4], whereas d-dimer is evaluated to exclude the diagnosis of venous thromboembolism [5,6]. For biomarkers to prove useful, they must be easy to measure, perform well as diagnostic tools, and exhibit some correlation with outcomes. Additionally, biomarkers can serve as surrogate markers in clinical trials. They have been incorporated into studies with the aim being to identify patients who might be eligible for certain experimental interventions and exclude those who are unlikely to benefit from a proposed novel treatment [7].

Severe sepsis and septic shock pose diagnostic challenges because many of the signs and symptoms in these conditions are nonspecific [8]. There is a pressing need to identify a biomarker that correlates with outcomes and that stratifies patients regarding the likelihood that they will benefit from novel therapies such as drotrecogin alfa (activated; DrotAA). Recently a sepsis definitions consensus conference [9] added specific biomarkers to the list of diagnostic criteria for sepsis.

Protein C (PC) is a vitamin K dependent plasma serine protease zymogen that is converted to activated PC by the thrombin-thrombomodulin complex. Activated PC has anticoagulant, anti-inflammatory, cytoprotective, and antiapoptotic activities [10-14].

PC deficiency is prevalent in severe sepsis, with studies showing that more than 80% of patients with severe sepsis have a baseline PC level below the lower limit of normal [15-18]. Unlike inflammatory cytokines, which are transiently elevated in severe sepsis, plasma PC levels decrease early in patients who develop severe sepsis, often before clinical symptoms appear, and these levels remain low initially but gradually rise in patients who recover and survive [18-21]. Numerous studies have examined the predictive value of plasma PC levels in sepsis [22-26]. Other studies have confirmed the association between depressed PC levels at baseline and the increased likelihood of adverse outcomes in sepsis, including time on a ventilator, time in the intensive care unit, development of shock, and increased mortality [17,18,20,21,25-33]. Previously reported placebo data from the PROWESS (Recombinant Human Activated Protein C Worldwide Evaluation in Severe Sepsis) trial showed that baseline PC levels and early changes in PC were prognostic of outcome. Change in PC levels on the first day after diagnosis of severe sepsis was highly correlated with outcome, with a decrease during the first days being able to differentiate eventual survivors from nonsurvivors [34]. However, broader reliance on PC as a biomarker in severe sepsis and septic shock requires evidence that serial changes over multiple time points provide valuable clinical information. Furthermore, it is necessary to demonstrate that measurement of PC provides information and insight not otherwise available from other biomarkers.

In order to validate the role of PC as a biomarker in severe sepsis and septic shock, we performed a secondary analysis of two large clinical trials of DrotAA. We compared the explanatory power of PC with those of multiple other clinical measures and biomarkers to determine the independent contribution that serial PC measurement would make in explaining mortality and DrotAA response.

## Materials and methods

### Patients

The PROWESS and ENHANCE (Extended Evaluation of Recombinant Activated Protein C) trials were conducted (before assignment of trial registration numbers) in accordance with ethical principles that have their origin in the Declaration of Helsinki and are consistent with good clinical practice and applicable laws and regulations. The trial designs, patient disposition, inclusion/exclusion criteria, and results were described previously [15,35]. PROWESS was a randomized, placebo-controlled clinical trial of DrotAA (Xigris^®^; Eli Lilly and Company, Indianapolis, IN, USA) in adult patients with severe sepsis. ENHANCE was an open-label, single-arm, clinical trial of DrotAA. All investigative sites obtained approval for the study from their institutional review board. Written informed consent was obtained from all patients or their legal representatives.

### Biomarker evaluations

In the PROWESS trial, plasma samples were obtained at baseline (day of randomization) and daily through to study day 7. A central laboratory (Covance Central Laboratory Services, Indianapolis, IN, USA) performed all assays. The PC activity assay was performed on a STA^® ^coagulation analyzer using the STA^®^-Staclot^® ^Protein C kit (Diagnostica Stago, Asnieres-Sur-Seine, France), which has a coefficient of variation of 7.5%. Protein S measurements were performed on the STA^® ^coagulation analyzer using the STA^®^- Staclot^® ^Protein S kit (Diagnostica Stago). Antithrombin III activity was quantitated using a chromogenic activity assay (Stachrome ATIII; Diagnostica Stago). IL-6 antigen levels were measured by enzyme immunoassay (Quantikine Human IL-6 HS kit; R&D Systems, Minneapolis, MN, USA). PC measurements during the ENHANCE trial were obtained at baseline and the end of infusion, and were analyzed using the same methodology as in PROWESS.

Sequential Organ Failure Assessment (SOFA) scores were determined based on local laboratory data, vasopressor dosages, and need for mechanical ventilation.

### Statistical methods

The statistical methods were designed to examine individually each laboratory and clinical measure for their attributes as biomarkers. Biomarkers have been classified into types by the National Institutes of Health Biomarker Definition Working Group [1]. Vasan [2] adapted the National Institutes of Health definitions to categorize biomarkers into type 0, 1, and 2; the definition of each type is given below. The following statistical tests examined each type of biomarker using data from the PROWESS trial. Data from the ENHANCE trial, in which all patients received DrotAA, were used to explore the consistency of findings; no combined analyses of the PROWESS and ENHANCE data were performed.

### Type 0 biomarker

A type 0 biomarker is, 'A marker of the natural history of a disease and correlates longitudinally with known clinical indices.' Initial analyses determined which of six laboratory measures and five organ dysfunctions (SOFA scores) were related at baseline to the clinical index of 28-day mortality in the placebo group. Based on placebo patients (n = 840), an 'optimal' cut-off was generated that maximized the sum of sensitivity and specificity (with each required to be at least 40%) to predict 28-day mortality. All values across the range of the receiver operating characteristic curves were examined. Using a cut-off for each measure allowed comparisons of odds ratios and interactions with treatment on a consistent binary scale across measures. In addition, these same measures at day 4 were evaluated for the placebo patients. Significance at both time points using the significance of χ^2 ^tests and 95% confidence intervals of odds ratios would indicate longitudinal correlation with mortality.

### Type 1 biomarker

A type 1 biomarker is, 'A marker that captures the effects of a therapeutic intervention in accordance with its mechanism of action.' This was examined in two ways for DrotAA in PROWESS. First, do more severe baseline values for the biomarker indicate a subgroup with a greater treatment benefit? This statistical interaction between biomarker and treatment was tested with Breslow-Day tests. The relative risks for death on comparing DrotAA (n = 850) with placebo (n = 840) were generated above and below cut offs. Second, biomarkers were identified that improved during treatment. Wilcoxon rank-sum tests were used to identify laboratory values and organ dysfunctions that were significantly different at day 4 between DrotAA and placebo patients. Day 4 last observation carried forward (LOCF) values were used in these analyses, with no imputation for death (the last observed SOFA score, not '4', was used for patients who died during the first 4 days). Patients with missing baseline values were excluded from these analyses.

### Surrogate end-point (type 2 biomarker)

A type 2 biomarker is, 'A marker that is intended to substitute for a clinical endpoint; a surrogate endpoint is expected to predict clinical benefit.' To quantify the potential of surrogate markers at the end of infusion, methods proposed by Li and coworkers [36] were utilized using Day 4 values. These methods use logistic regression to provide the 'proportion of treatment effect explained' (PTEE). PTEE has been proposed as a measure of surrogacy for the validation of surrogate end-points. A good surrogate marker accounts for a larger percentage of treatment effect. For instance, if a treatment reduces the risk for death by 20% and improvement in a biomarker was associated with a risk reduction of death by 10%, then the biomarker explains 50% of the treatment effect. This was quantified by taking the ratio of risk reduction explained solely by the average change in a measure, and dividing by total risk reduction associated with the average change in a measure plus the residual treatment effect. These analyses were to determine how much of the 28-day mortality effect was accounted for solely by patient status on day 4. The PTEE values of the multiple variables examined are not expected to add up to 100%, and a negative PTEE means that the treatment resulted in a change in the variable that is in the opposite direction than anticipated for a beneficial treatment effect.

### Additional statistical methods

Additional nonparametric analyses were performed using Wilcoxon sign-rank and Wilcoxon rank-sum tests, as appropriate. All calculations were performed using SAS version 8.1 software (SAS Institute Inc., Cary, NC, USA).

## Results

The baseline characteristics for the PROWESS placebo and DrotAA patient populations have been reported elsewhere [15], as have those of the ENHANCE population [35]. However, a summary of selected baseline characteristics that are specifically relevant to the present analyses is given in Table 1.

**Table 1:** PROWESS and ENHANCE patient baseline characteristics

Variable	PROWESS		ENHANCE
	Placebo (n = 840)	DrotAA (n = 850)	DrotAA (n = 2378)
Sex (% male [*n*])	58.0 (487)	56.1 (477)	58.2 (1383)
Mean age (years [SD])	60.6 (16.5)	60.5 (17.2)	59.1 (16.9)
Caucasian (% [*n*])	82.0 (689)	81.8 (695)	90.6 (2154)
APACHE II score (mean [SD])	25.0 (7.8)	24.6 (7.6)	22.0 (7.4)
SOFA score (mean [SD])			
Cardiovascular	2.7 (1.5)	2.6 (1.5)	3.0 (1.4)
Respiratory	2.7 (1.1)	2.7 (1.0)	2.7 (1.0)
Renal	1.1 (1.1)	1.1 (1.1)	1.3 (1.2)
Hematologic	0.7 (1.0)	0.7 (0.9)	0.8 (1.0)
Hepatic	0.6 (0.9)	0.6 (0.8)	0.7 (0.9)
Protein C (median [IQR])	50 (33 to 68)	47 (30 to 63)	45 (30 to 64)
Protein S (median [IQR])	38 (23 to 58)	35 (33 to 57)	-
Antithrombin III (median [IQR])	60 (45 to 75)	58 (43 to 74)	-
Interleukin-6 (median [IQR])	484 (129 to 2539)	496 (153 to 2701)	-
Prothrombin time (median [IQR])	18.6 (16.4 to 21.8)	18.7 (16.6 to 22.1)	-
D-dimer (median [IQR])	4.1 (2.2 to 8.7)	4.2 (2.3 to 8.1)	-

APACHE, Acute Physiology and Chronic Health Evaluation; DrotAA, drotrecogin alfa (activated); ENHANCE, Extended Evaluation of Recombinant Activated Protein C; PROWESS, Recombinant Human Activated Protein C Worldwide Evaluation in Severe Sepsis; SD, standard deviation; SOFA, Sequential Organ Failure Assessment; IQR, interquartile range.

### Type 0 biomarker: relationship to natural history of sepsis and correlated with clinical outcome

Baseline values of six laboratory and five clinical measures were evaluated as potential predictors of 28-day mortality. To allow comparisons across measures, the cut-off values associated with greater risk for mortality based on sensitivity and specificity analyses of baseline values were determined (Table 2). The number of patients at increased risk based on the cut-offs, although each representing a different subgroup, was very similar across variables, representing approximately one-third of patients. However, this does not represent the same high-risk patients in each group. Only one patient was high risk for all 11 markers, and only 48 (5.8%) were low risk for all of their measures. This approach established a baseline PC level < 40% as a useful end-point for assessing mortality risk in sepsis patients. The odds of dying within 28 days was twice as high in patients with a baseline PC level < 40% as in those with a PC level of ≥40%. Similar odds ratios were associated with the cut-off values of the other variables, as were the areas under the receiver operating characteristic curve, a combined measure of sensitivity and specificity. This analysis also demonstrates (as already known) the unequal effect of individual SOFA scores, with cut-off ranging from ≥1 for renal SOFA to ≥4 for cardiovascular and respiratory SOFA.

**Table 2:** Relationship of baseline (start of infusion) values to 28-day mortality in PROWESS placebo patients

Baseline measure	Cut-off^a^	Number of patients at increased risk using cut-off (n [%])	Odds ratio (95% CI)	AUC^b^
Protein C (%)	<40	243 (31.4%)	2.12 (1.55–2.89)	58.9%
Protein S (%)	<46	239 (31.5%)	1.91 (1.38–2.64)	57.7%
Antithrombin III (%)	<53	240 (31.4%)	2.32 (1.70–3.18)	60.1%
interleukin-6 (pg/ml)	≥704.6	252 (31.2%)	2.21 (1.63–2.99)	59.7%
Prothrombin time (seconds)	≥18.4	240 (31.5%)	1.89 (1.38–2.58)	57.4%
D-dimer (μg/ml)	≥4.45	241 (31.8%)	1.51 (1.11–2.05)	55.1%
Cardiovascular SOFA	≥4	259 (30.8%)	1.63 (1.21–2.18)	56.0%
Respiratory SOFA	≥4	257 (31.2%)	1.76 (1.27–2.44)	55.5%
Renal SOFA	≥1	258 (30.8%)	2.14 (1.55–2.95)	58.6%
Hematologic SOFA	≥2	259 (30.8%)	1.69 (1.20–2.38)	54.6%
Hepatic SOFA	≥2	239 (31.3%)	1.31 (0.89–1.93)	52.1%

^a^Cut-off based on maximum sensitivity and specificity when both were ≥ 40% for predicting 28-day mortality. Using a cut-off for each measure allowed comparison of odds ratios and treatment interactions on a consistent binary scale across variables. ^b^Area under the receiver operating characteristic curve (AUC) based on 28-day mortality outcome in logistic regression models with the cut-off as the univariate independent variable; this is a combined measure of sensitivity and specificity. CI, confidence interal; PROWESS, Recombinant Human Activated Protein C Worldwide Evaluation in Severe Sepsis; SOFA, Sequential Organ Failure Assessment.

To determine which measures exhibited a longitudinal correlation with mortality, these same measures were evaluated at the end-of-infusion period (day 4) for placebo patients. The optimal cut-off values at day 4, shown in Table 3, were very similar to those shown for baseline values in Table 2, except that the cut-off value for IL-6 was ≥185.6 versus ≥704.6 pg/ml. Without adjusting for baseline values, the day 4 values for all 11 variables were associated with a statistically significant increased risk for death at day 28.

**Table 3:** Relationship of day 4 (end of infusion) values to 28-day mortality in PROWESS placebo patients

		Sample size		Univariate odds ratio	
		
Measure	Cut-off^a^	Higher risk	Lower risk	Odds ratio (95% CI)	*P*
Protein C (%)	<42	240	578	4.63 (3.35 to 6.41)	<0.0001
Protein S (%)	<42	297	521	3.25 (2.38 to 4.43)	<0.0001
Antithrombin III (%)	<60	306	512	4.17 (3.05 to 5.71)	<0.0001
Interleukin-6 (pg/ml)	≥185.6	264	563	7.27 (5.23 to 10.11)	<0.0001
Prothrombin time (seconds)	≥18.4	233	585	6.13 (4.40 to 8.56)	<0.0001
D-dimer (μg/ml)	≥4.63	408	410	2.13 (1.57 to 2.89)	<0.0001
Cardiovascular SOFA	≥3	301	539	7.32 (5.28 to 10.13)	<0.0001
Respiratory SOFA	≥3	358	482	3.36 (2.48 to 4.56)	<0.0001
Renal SOFA	≥1	335	505	4.82 (3.52 to 6.59)	<0.0001
Hematologic SOFA	≥2	252	588	3.42 (2.50 to 4.68)	<0.0001
Hepatic SOFA	≥1	256	573	2.11 (1.55 to 2.89)	<0.0001

^a^Cut-off was defined by the day 4 (end of infusion) value that resulted in maximum sensitivity and specificity for predicting 28-day mortality. CI, confidence interal; PROWESS, Recombinant Human Activated Protein C Worldwide Evaluation in Severe Sepsis; SOFA, Sequential Organ Failure Assessment.

### Type 1 biomarker: therapeutic intervention in accordance with mechanism of action

**Figure 1:**
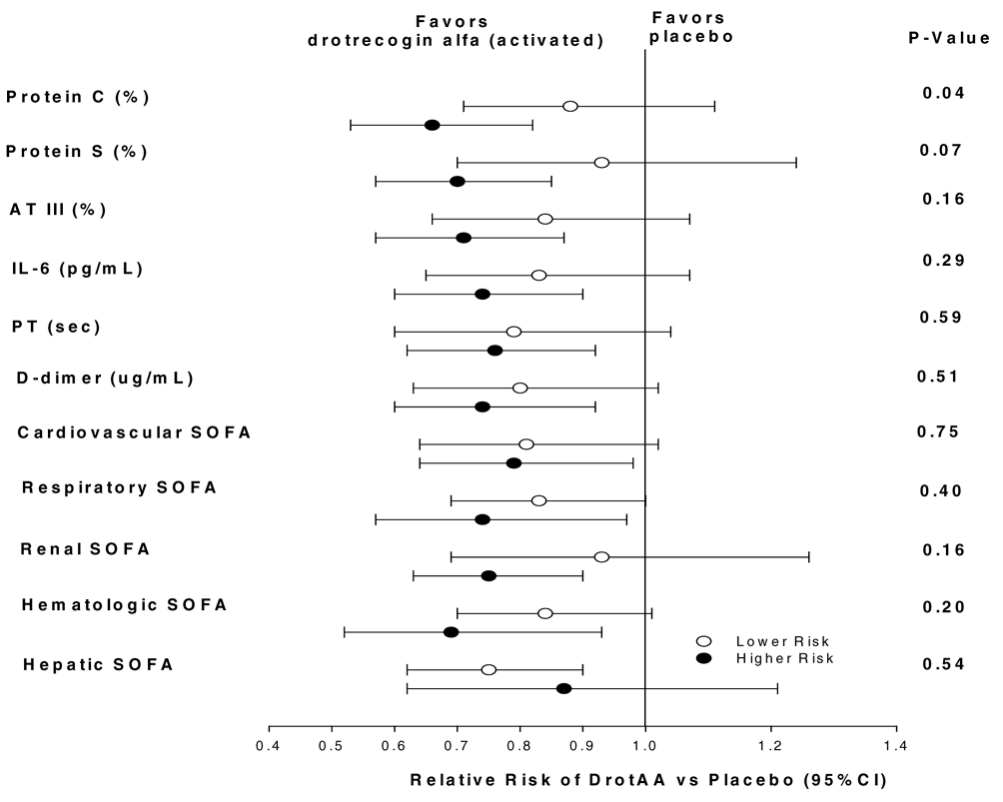
Illustration of 28-day mortality RR reduction (DrotAA versus placebo) for each potential biomarker at baseline. The point estimates of relative risk (RR) for death in patients at lower risk and higher risk, based on statistically defined cut-offs (shown in Table 2), are indicated by open ovals and solid ovals, respectively; 95% confidence intervals (CIs) are indicated by horizontal lines. Only protein C (PC) was significantly (*P *< 0.05) different between the two risk groups, as indicated by the least overlap in CIs, indicating a differential benefit. *P *values were determined using Breslow-Day tests. AT, antithrombin; CI, confidence interval; DrotAA, drotrecogin alfa (activated); IL, interleukin; PT, prothrombin time; SOFA, Sequential Organ Failure Assessment.

Figure 1 shows the therapeutic effect of DrotAA in patients at lower and higher risk for death, as defined by the statistically defined baseline cut-off for the 11 potential biomarkers shown in Table 2. PC was the only biomarker at baseline that exhibited a statistically significant difference in relative risk for death between the lower and higher risk groups (relative risk 0.66 [DrotAA and placebo] for lower risk versus 0.88 for higher risk patients; *P *= 0.04). In PROWESS, patients who had values below the PC cut-off (< 40%) and who were administered DrotAA exhibited a 34% reduction in risk for death (27.6% DrotAA versus 41.8% placebo), whereas above the cut-off the risk reduction was 12% (22.4% versus 25.3%). Mortality rates observed in the ENHANCE trial were 33.3% for patients with PC below the cut-off and 17.6% for those with PC above the cut-off (data not shown).

### Surrogate endipoint (type 2 biomarker): substitute for clinical end-point by predicting clinical benefit

The next step was to determine which of the potential biomarkers improved during DrotAA treatment (Table 4). In PROWESS, at the end of the 4-day infusion (day 4) DrotAA significantly increased the median PC level (*P *< 0.0001), increased prothrombin time (*P *= 0.0003) and decreased d-dimer (*P *< 0.0001), and, to a lesser degree, decreased the cardiovascular SOFA score (*P *= 0.01) and increased the hepatic SOFA score (*P *= 0.04). Although all of these post-baseline measures were prognostic for placebo mortality (Table 3), the end of infusion (day 4 LOCF) level of PC and, to a lesser degree, cardiovascular dysfunction and d-dimer appeared to be specifically improved with DrotAA treatment. In PROWESS, the median increase in PC activity during the 4-day infusion period was 19% (interquartile range [IQR] 3% to 36%) for DrotAA, as compared with 8% (IQR -5% to +25%) for placebo patients. In the same timeframe, ENHANCE patients receiving DrotAA exhibited an 18% increase in PC (IQR 0% to 39%). Because the negative relationship of DrotAA treatment with hepatic SOFA on day 4, we reviewed the actual baseline and day 4 bilirubin measurements. Considering the 1,374 patients in PROWESS for whom data were available regarding the change in bilirubin from baseline at these time points (n = 692 for DrotAA and n = 682 for placebo), there were no significant changes within groups (*P *= 0.49 for DrotAA and *P *= 0.12 for placebo) or between therapies (*P *= 0.14), with a median change of 0 and -1 μmol/l for DrotAA and placebo, respectively.

**Table 4:** Day 4 (end of infusion) values in PROWESS:individual surrogate performance score (PTEE)

Day 4 measure	DrotAA patients (mean [SD]/median)	Placebo patients (mean [SD]/median)	*P*^a^	Individual surrogate performance score (PTEE)^b^
Protein C (%)	70.6 (36.2)/67.0	62.7 (36.9)/59.0	<0.0001	57.2%
Protein S (%)	53.3 (29.0)/52.0	53.5 (30.2)/53.0	0.95	-0.8%^c^
Antithrombin III (%)	70.3 (27.0)/69.5	69.0 (28.0)/70.0	0.38	13.4%
Interleukin-6 (pg/ml)	3,649 (30,280)/75.5	4948 (33379)/79.5	0.78	0.3%^d^
Prothrombin time (seconds)	18.6 (7.4)/16.7	18.5 (8.4)/16.2	0.0003	-30.4%^c, d^
D-dimer (μg/ml)	4.63 (5.41)/3.12	7.13 (7.87)/4.61	<0.0001	40.5%^d^
Cardiovascular SOFA	1.48 (1.60)/1.00	1.67 (1.64)/1.00	0.01	40.8%
Respiratory SOFA	2.25 (1.03)/2.00	2.30 (1.01)/2.00	0.25	12.7%
Renal SOFA	0.74 (1.12)/0.00	0.80 (1.17)/0.00	0.39	10.6%
Hematologic SOFA	0.89 (1.12)/0.00	0.92 (1.15)/0.00	0.79	5.1%
Hepatic SOFA	0.61 (0.92)/0.00	0.53 (0.89)/0.00	0.04	-13.6%

^a^*P *value for Wilcoxon rank-sum test. ^b^The performance score shows the proportion of drotrecogin alfa (activated; DrotAA) treatment effect explained (PTEE) based on logistic regression analyses that quantify the amount of the observed treatment effect on 28-day mortality that is attributable to the treatment effect of the individual biomarker. Because changes in these biomarkers are often interdependent (for example, protein C and cardiovascular SOFA improvements), the performance scores are not expected to add up to 100%. ^c^A negative performance score means that the treatment adversely affected the variable. ^d^For biomarkers that varied greatly between mean and median values, analysis was based on median value. PROWESS, Recombinant Human Activated Protein C Worldwide Evaluation in Severe Sepsis; SD, standard deviation; SOFA, Sequential Organ Failure Assessment.

To assess how the change helps to account for the DrotAA treatment effect in PROWESS, the PTEE was analyzed (Table 4). The end-of-infusion (day 4 LOCF) measure of PC accounted for 57.2% of the DrotAA treatment effect on 28-day mortality. The change in prothrombin time and the hepatic SOFA, while being statistically different between treatment groups, exhibited a negative correlation with the DrotAA treatment effect (namely, its direction was opposite that anticipated for a beneficial treatment effect).

### Further examination of protein C and DrotAA interactions over time

**Figure 2:**
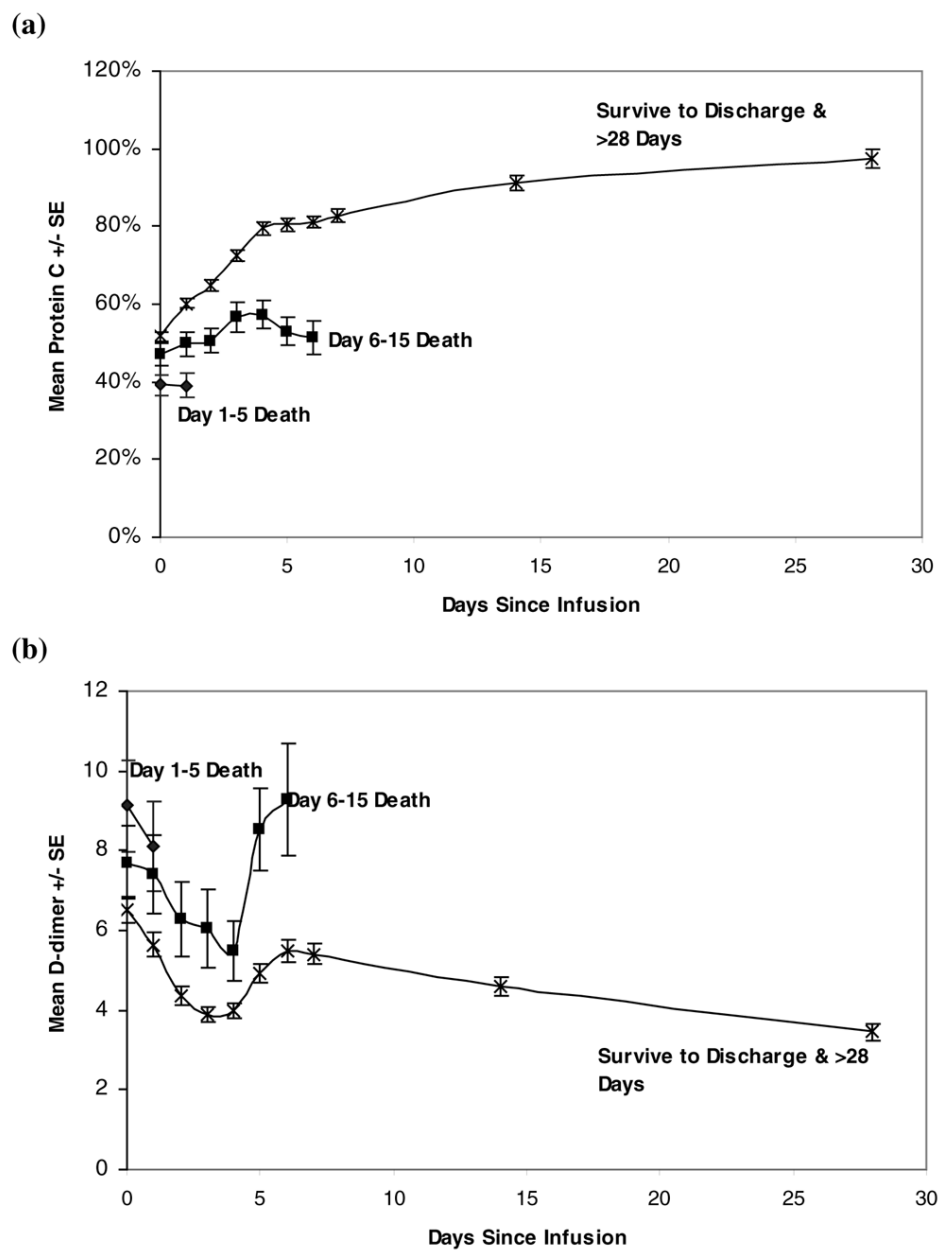
PC and d-dimer levels. Shown are the mean ± standard error **(a) **protein C (PC) and **(b) **d-dimer levels based on time of death. Raw values with no imputation were included. PROWESS (Recombinant Human Activated Protein C Worldwide Evaluation in Severe Sepsis) drotrecogin alfa (activated; DrotAA) patients with baseline measures were classified according to timing of death (n = PC/d-dimer): death ≤ 5 days after start of infusion (n = 79/86); death after 6 to 15 days (n = 81/84); and survival to day 28 and hospital discharge (n = 544/577). The PC data were reported by Vangerow and coworkers [42] and comparable PC data for PROWESS placebo patients were reported by Macias and Nelson [22].

A strong link between improved PC levels and improved survival became apparent when serial PC levels were analyzed for DrotAA treated patients and displayed by time of death or ultimate survival (Figure 2a). (A similar figure for the PROWESS placebo patients was previously reported [22].) As with the PROWESS placebo patients, PC levels assessed at the start of each time interval were highly predictive of outcome within the time interval, with continued PC deficiency associated with higher mortality. This analysis demonstrates that patients with lower PC levels are more likely to die, and that patients who survived to be discharged had PC levels that increased during the DrotAA infusion to a mean of 80% by day 5. Patients who died between days 6 and 15 had a decrease in mean PC levels after day 4, suggesting that PC levels fell when DrotAA infusion was stopped.

Based on the statistical analyses presented in Table 4, d-dimer values also appeared to be a potential surrogate biomarker for DrotAA therapy. However, as shown in Figure 2b, although d-dimer decreased in all patients who received DrotAA, at the end of the infusion the d-dimer levels immediately began to increase in all patients and that increase was not correlated with mortality at different time points.

### Summary of results correlated with biomarker status

To aid in the interpretation of these data, the results are summarized in Table 5 using categories defined in the footnote. This summary shows that PC is the only biomarker that consistently correlated with outcome, regardless of the time of measurement or the analytical approach.

**Table 5:** Summary of results in support of biomarker status

	Type 0 biomarker: placebo baseline value versus mortality (see Table 2); categorized by OR^a^	Type 0 biomarker: placebo day 4 value versus mortality (see Table 3); categorized by *P *value^b^	Type 1 biomarker: relationship of baseline value to DrotAA effect (see Figure 1); categorized by *P *value^b^	Surrogate (type 2 biomarker): improvement at day 4 with DrotAA (see Table 4); categorized by *P *value^b^	Surrogate (type 2 biomarker): surrogate performance score (see Table 4); categorized by PTEE^c^
Protein C	+++	+++	++	+++	+++
Protein S	++	+++	+	-	-
Antithrombin III	+++	+++	-	-	+
Interleukin-6	+++	+++	-	-	-
Prothrombin time	++	+++	-	+++	-
D-dimer	++	+++	-	+++	++
Cardiovascular SOFA	++	+++	-	++	++
Respiratory SOFA	++	+++	-	-	+
Renal SOFA	+++	+++	-	-	+
Hematologic SOFA	++	+++	-	-	+
Hepatic SOFA	+	+++	-	+	-

Shown is the categorization based on the results of each analysis. To summarize the statistical analyses, the results from each analysis were categorized as follows. ^a^Odds ratios (ORs) from Table 2: - = OR < 0; + = 0 ≤ OR < 1.5; ++ = OR 1.5 to 2.0; +++ = OR > 2.0. ^b^*P *values from Tables 3 and 4, and Figure 1: - = *P *> 0.1; + = 0.051 <*P *≤ 0.1; ++ = *P *0.01 to 0.05; +++ = *P *< 0.01. ^c^Proportion of treatment effect explained (PTEE) from Table 4: - = negative or < 5%; + = 5% to < 25%; ++ = 25% to 50%; +++ = > 50%. DrotAA, drotrecogin alfa (activated); SOFA, Sequential Organ Failure Assessment.

**Figure 3:**
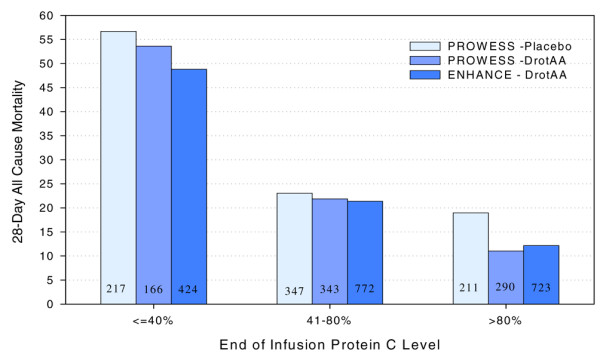
Mortality from PROWESS and ENHANCE based on end-of-infusion PC levels by categories. The protein C (PC) categories were normal (> 80%), deficient (41% to 80%), and severely deficient (< 40%). The number in each column is the total number of patients in each category. Patients were included if they had a baseline PC measure. Day 4 PC was classified as end of infusion. If day 4 measurement was not available, last observation carried forward values were used for classification. These data were reported by Vangerow and coworkers [42]. ENHANCE, Extended Evaluation of Recombinant Activated Protein C; PROWESS, Recombinant Human Activated Protein C Worldwide Evaluation in Severe Sepsis.

Figure 3 shows that PC levels at end of infusion also correlated with outcome regardless of treatment. For this final analysis, end-of-infusion (day 4 LOCF) PC levels were categorized by deficiency (severe ≤40%, moderate 41% to 80%, or normal > 80%) and the categories were shown to be significantly related to mortality regardless of treatment. DrotAA treatment resulted in fewer patients (166 [20.8%] versus 217 [28.0%]) with severe PC deficiency (≤40%), and more patients (290 [36.3%] versus 211 [27.2%]) with normal PC levels (> 80%) at the end of infusion compared with placebo (*P *< 0.0001). The ENHANCE mortality rates based on day 4 PC categories were consistent with the PROWESS DrotAA data. Regardless of treatment, mortality rates were lowest in patients with normalized PC levels. The histograms within each PC category appeared equal across treatments, indicating that differences between treatment groups do not exist after taking into account the surrogate end-point. The end-of-infusion PC category has a greater effect on mortality than differences within a category between DrotAA and placebo treatment.

## Discussion

These analyses indicate that PC can act as a surrogate end-point in severe sepsis, regardless of the time of measurement or treatment received. Based on a systematic statistical assessment of six potential biomarkers and five organ dysfunction measures that were measured in a large randomized clinical trial conducted in severe sepsis patients, only PC levels were significantly correlated with 28-day mortality regardless of statistical approach. Using statistically defined cut-offs, multiple variables at baseline were predictive of 28-day mortality and all variables could be predictive at the end of the 4-day infusion. Only PC improved with DrotAA treatment, was significantly correlated with the DrotAA treatment effect, and accounted for more than 50% of its treatment effect (PTEE). Additionally, serial changes in PC correlated well with mortality. Variations in PC also explained the majority of the treatment effect due to DrotAA therapy.

Other biomarkers have been proposed to be useful diagnostic markers for sepsis and the severity of sepsis [37], but those analyzed in this study did not meet all criteria for surrogacy. D-dimer did decrease during infusion with DrotAA and, based on PTEE analysis, it could account for 41% of its treatment effect, but there was no difference in relative risk between the lower and higher risk groups. At the end of infusion the values increased in all patients, both survivors and nonsurvivors, suggesting that the DrotAA effect is not just an alteration in the procoagulant state. Instead, based on PROWESS data, d-dimer appears to be a marker that the patient received DrotAA, not how well the patient responded to DrotAA. IL-6 levels at baseline were predictive of patient outcome, but at the end of infusion (day 4) the difference between DrotAA and placebo groups was not significant and its PTEE was 0.3%. Prothrombin is another biomarker that is expected to decrease as coagulopathy improves, but instead prothrombin time increased slightly, but consistently, in patients who received DrotAA, resulting in a negative PTEE value (-30%). Although DrotAA improves coagulopathy, there is also a small direct effect of interference of activated PC with prothrombin time. The negative PTEE obtained for hepatic SOFA (-14%) results from a slightly higher hepatic SOFA with DrotAA than with placebo. However, there were no significant differences in change in bilirubin from baseline to day 4. Traditionally, liver dysfunction under conditions of shock and sepsis is considered to be biphasic, with an initial ischaemic insult (ischaemic hepatitis) followed by jaundice (intensive care unit jaundice) developing several days later [38,39]. Thus, the SOFA subscore for hepatic impairment, which is based on bilirubin levels, is biased toward underestimation of dysfunction in the early course of the disease.

Some have expressed skepticism toward PTEE analyses, especially when it is applied to small individual studies, because high PTEE values do not necessarily imply that the surrogate end-point is an important part of the causal pathway that leads from treatment to disease [40]. However, our analyses are based on a large population (n = 1,690) and show that PC levels not only can explain a large proportion of the DrotAA treatment effect but also are directly related to clinical outcome in severe sepsis.

PC meets the US National Institutes of Health's recommended definition of a biomarker that could function as a clinical end-point (a variable that reflects how long or how a patient feels or functions, or how long a patient survives), as well as a surrogate end-point (a biomarker, based on epidemiologic, therapeutic, pathophysiologic, or other scientific evidence, intended to substitute for a clinical end-point) [40]. Surrogate end-points can be useful in advising patients about modifications of treatment after they have reached a surrogate end-point but have not yet reached the true clinical end-point. Our data shows PC to be a valid surrogate, defined as a biomarker that can explain at least 50% of the effect of an exposure or intervention on the outcome of interest [41]. Of the biomarkers analyzed, only PC had a PTEE greater than 50%. The PTEE for cardiovascular SOFA was 41%. PC predicts cardiovascular changes downstream, and so it is expected that the cardiovascular SOFA would be a reasonable surrogate. However, the reverse is not true; cardiovascular improvement does not necessarily increase PC downstream, and the baseline cardiovascular SOFA does not appear to predict well who will benefit most from DrotAA treatment. Although this is somewhat counterintuitive, it is what the PROWESS data have indicated.

Normalization of PC levels is critical for survival. The serial measurements of PC show that if the PC values continue to increase toward normalization after day 4, then survival increases. For those patients who died between days 6 and 15, the PC levels were increasing until after the end of drug infusion, which raises the question of whether these patients would have survived if the DrotAA infusion had been extended beyond day 4. Use of PC levels to optimize therapy for individual patients warrants further study. A series of studies are proposed to explore the use of serial plasma PC measurement as a biomarker that will achieve the following objectives: aid in the identification of patients with severe sepsis who are most likely to benefit from DrotAA; enable the adjustment of DrotAA therapy for individual patients (specifically, the possibility to use a higher dose and to adjust the infusion duration, making it either longer or shorter as needed); and provide guidance to the clinician regarding whether the patient is responding to DrotAA. The first study in the series is referred to as RESPOND (Research Evaluating Serial PC Levels in Severe Sepsis Patients on DrotAA) and is currently ongoing. It is seeking to demonstrate that 'alternative therapy' (higher dose with variable infusion duration or variable infusion duration only) results in a greater increase in PC levels than 'standard therapy' (the currently approved regimen of 24 μg/kg per hour for 96 hours) and, importantly, to provide appropriate safety and efficacy data to determine the most appropriate aspects of 'alternative therapy' to incorporate into possible future studies [42].

### Limitations

This was a *post hoc *analysis that was limited to the potential biomarkers measured during PROWESS. When PROWESS was designed the prevailing assumed mechanism of action of PC was anticoagulation, and so the laboratory measurements in that study focused primarily on the coagulation pathway.

Many of the potential biomarkers included in our analyses do not have prespecified clinically defined thresholds. Therefore, to be consistent in how the variables were analyzed, statistically defined cut-offs were determined from specificity and sensitivity analyses. The cut-offs were driven by variability within the patient population in PROWESS and were therefore limited by a one-study dataset. We used ENHANCE data in an attempt to validate our findings, but that comparison is not ideal because ENHANCE had no placebo group and PC was measured less frequently during the trial. Also, the areas under the receiver operating characteristic curves for all markers tended to be at the 60% level or below. In the PROWESS population, in which the extremes of risk for death are excluded by inclusion and exclusion criteria, individual markers of baseline severity have relatively low values for prognostic measures in univariate analyses.

Finally, in an attempt to put our analyses into perspective and to help summarize the results from the different analyses, we arbitrarily assigned categories to the outcomes, as shown in Table 5. This was an effort to illustrate, not quantitate, the results.

## Conclusion

Based on systematic analyses of 11 variables (six biomarkers and five organ dysfunctions) measured in severe sepsis clinical trials, PC was the only variable consistently correlated with both DrotAA treatment effect and survival. Further study is needed to determine whethter longer infusions or higher doses of DrotAA would achieve the goal of normalizing PC in more patients with severe sepsis.

## Key messages

• Serial measurement of PC in sepsis has the potential to act as a biomarker to predict outcome and guide therapy with DrotAA.

• Based on systematic analyses of 11 variables (six biomarkers and five organ dysfunctions) measured in severe sepsis clinical trials, PC was the only variable consistently correlated with both survival and DrotAA treatment effect.

• A PC level < 40% was established as a useful predictor of outcome at baseline and at the end of infusion.

• Normalization of PC levels is an important predictor of survival, and DrotAA treatment results in more patients with normal PC levels and fewer patients with severe PC deficiency at the end of infusion compared with placebo.

• Further study is needed to determine whether longer infusions or higher doses of DrotAA would achieve the goal of normalizing PC in more patients with severe sepsis.

## Abbreviations

DrotAA = drotrecogin alfa (activated); ENHANCE = Extended Evaluation of Recombinant Activated Protein C; IL = interleukin; IQR = interquartile range; LOCF = last observation carried forward; PC = protein C; PROWESS = Recombinant Human Activated Protein C Worldwide Evaluation in Severe Sepsis; PTEE = proportion of treatment effect explained; SOFA = Sequential Organ Failure Assessment.

## Competing interests

DRN, JJ, and GMV are employees of and stockholders in Eli Lilly and Company (Eli Lilly), the manufacturer of DrotAA. AFS is a consultant to both Eli Lilly and Astra Zeneca regarding the design of clinical trials for severe sepsis and septic shock. DLAW has given paid lectures for and participated in clinical trials, supported by Eli Lilly. KR has served as consultant and received payments from Eli Lilly for speaking engagements and research. FB received payments from Eli Lilly for speaking engagements and research.

## Authors' contributions

AFS, DRN, and JJ participated in the conception and design of the study. AFS, and DLAW participated in the clinical trials and data collection. GMV participated in the conception of the study. All authors contributed to the development and conduct of analyses, and participated in drafting the manuscript. All authors contributed to revisions and approval of the final manuscript.
